# Effects of Aging on Kidney Graft Function, Oxidative Stress and Gene Expression after Kidney Transplantation

**DOI:** 10.1371/journal.pone.0065613

**Published:** 2013-06-18

**Authors:** Rui Ding, Xiangmei Chen, Di Wu, Ribao Wei, Quan Hong, Suozhu Shi, Zhong Yin, Linlin Ma, Yuansheng Xie

**Affiliations:** State Key Laboratory of Kidney Disease, Department of Nephrology, Chinese PLA General Hospital, Beijing, P. R. China; The Ohio State University, United States of America

## Abstract

Conflicting results have been reported regarding the effects of donor age, recipient age and donor-recipient age difference on short- and long-term outcomes after kidney transplantation. The aim of this study was to evaluate the effects of recipient age on graft function, oxidative stress, and gene expression after renal transplantation. Fifty male Fischer 344 rats [25 young (Y, 4 months), 25 senior (S, 16 months)] were randomized to 6 groups: 2 sham groups (Y and S, n = 5 in each group) and 4 renal transplant groups[young-to-young (Y-Y), young-to-senior (Y-S), senior-to-young (S-Y), senior-to-senior (S-S), (n = 10 in each group)]. The left kidneys were transplanted from donor to recipient. After 12 weeks, systematic blood pressure, graft weight, graft function, histology and oxidative stress were measured. Microarray analysis and quantitative real-time PCR confirmation were performed to study gene expression in the grafts. There were no differences in renal graft function between young and senior kidney cross-transplantation. Transplanted kidneys showed no significant differences in glomerulosclerosis index compared to non-transplanted kidneys but had significantly different tubulointerstitium scores compared to age-matched controls. Senior rats had lower SOD activity and higher MDA content than young rats. SOD activity was significantly lower and MDA content significantly higher in the Y-S group than in the Y-Y group. There were 548 transcript differences between senior and young kidneys with 36 upregulated and 512 downregulated transcripts. There were 492 transcript differences between Y-S and Y-Y groups with 127 upregulated and 365 downregulated transcripts. There were 1244 transcript differences between the S-Y and S-S groups with 680 upregulated and 574 downregulated transcripts. Oxidative stress and gene expression profile was significantly different in the Y-S compared to the S-Y group. The identified differences were mainly in the MAPK and insulin signal pathways, making these potential targets for therapeutic intervention.

## Introduction

Kidney transplant (KT) donors [1] and recipients [2] are now increasingly elderly. The growing numbers of patients with end-stage kidney disease, and improvements in short-term KT outcomes, have increased the number of patients who are at risk of the long-term complications of KT. Despite improvements in KT techniques, whether and how donor and recipient age affect graft function and patient survival after KT remain debatable. Conflicting results have been reported regarding the effects of donor age [3]–[6], recipient age [7], [8] and donor-recipient age difference [9], [10] on short- and long-term outcomes after KT.

Kidneys are known to be affected by the aging progress. Oxidative stress may be the most important cause of aging and aging-related disease according to the “double-agent” aging theory [11]. The contribution of oxidative stress to the development of aging may be a sort of double jeopardy for outcomes after KT because older recipients of renal allografts have reduced anti-oxidative capacity, which may be associated with poorer outcome [12]. If transplanted kidneys age at an accelerated rate relative to other organs in the recipient, slowing or reversing this process may be a useful strategy to improve outcomes after KT. Indeed, reduced oxidative damage, as shown by reduced levels of oxidation and apoptosis, at 6 months after transplantation correlated with a better recovery of renal function in kidney allografts [13].

In terms of kidney aging, genetic factors may influence tissue damage and the related loss of function in elderly recipients [14]. Gene expression profiling using microarrays or quantitative PCR has become a benchmark in research into novel and informative monitoring assays for KT [15]. Profiling gene expression would allow modification of post-transplant management and, thereby, potentially improve short- and long-term KT outcomes.

The aim of this study was to determine how recipient age affects oxidative stress, graft function and gene expression. We performed kidney cross-transplantation experiments in inbred rats to investigate the effects of artificially accelerated or delayed aging on the grafted kidney in the absence of inheritance and immuno-rejection effects. To avoid any effects of long-term ischemia/reperfusion injury [16], a 12-week-long kidney cross-transplantation experiment between young and senior Fischer 344 rats was performed.

## Materials and Methods

### Ethics Statements

This study was carried out in strict accordance with the recommendations in the Guide for the Care and Use of Laboratory Animals of the National Institutes of Health. The protocol was approved by the Committee on the Ethics of Animal Experiments of PLA General Hospital, Beijing, China (Permit Number: 2009-X4-15). All surgery was performed under sodium pentobarbital anesthesia, and all efforts were made to minimize suffering.

### Animal Model

Experiments were performed on 50 male Fischer 344 (Vital River Co. Beijing, China) rats (25 young [Y, 4 months, 267.9+/−15.4 g]; 25 senior [S, 16 months, 470.6+/−16.8 g]), we chose this kind of rat because they were an inbred strain of brothers and sisters after 20 generations without immune rejection after KT. Sham-operated rats were used as controls. Syngeneic kidney transplantation was performed between 40 male Fischer 344 rats, using the following groups (n = 10 in each group): kidney transplants from young to senior rats (Y-S group); kidney transplants from young to young rats (Y-Y group); kidney transplants from senior to young rats (S-Y group); and kidney transplants from senoir to senior rats (S-S group). The left donor kidney was perfused with 25 U/mL of cold heparin in saline, and stored at 4°C prior to orthotopic transplantation. Body temperature was maintained between 36°C and 38°C during the operation. The right native kidneys of the recipients were not removed to avoid compensatory hypertrophy of the grafted kidney. Grafted kidney weight was determined before transplantation and at the end of the experiment, and systolic blood pressure was measured monthly by the tail cuff method. All rats were sacrificed at the end of 12 weeks. Kidney tissues were snap-frozen in liquid nitrogen and stored at –70°C for transcriptional studies, fixed in formaldehyde for histopathological assessment.

### Assessment of Renal Function

The glomerular filtration rate (GFR) was measured by the clearance of 99mTc-DTPA at the end of 12 weeks. The rats were anesthetized with 2% pentobarbital sodium (35 mg/kg) and placed under the probe of a Symbia T Radionuclide scanner (Siemens, Bonn, Germany); 1 mCi of 99mTc-DT PA was injected intravenously using an insulin syringe. The grafted kidney GFR was calculated using the Gates formula [17].

### Histological Examination

Formaldehyde-fixed and paraffin-embedded sections of the kidney were cut at a thickness of 2 µm, and stained with periodic acid Schiff (PAS). Age-related renal changes were assessed histopathologically in glomeruli and the tubulointerstitium in a blinded manner by two experienced renal pathologists who were unaware of the animal groups. Glomerulosclerosis was expressed as the percentage of globally sclerosed glomeruli based on all glomeruli present in the section. Tubulointerstitial scores were used to evaluate tissue damage: a numerical score was used to define the degree of tubular cell damage: 0, no damage; 1, unicellular, patchy, isolated necrosis; 2, tubular necrosis less than 25%; 3, tubular necrosis between 25 and 50% and 4, tubular necrosis more than 50% and presence of infracted tissue.

### SA-β-galactosidase Staining

Frozen sections were cut at a thickness of 4 µm, dried for 15 min at room temperature, and then washed in PBS. Sections were fixed for 5 min in 2% formaldehyde/0.2% glutaraldehyde at room temperature. The slides were incubated overnight at 37°C with fresh SA-β-gal stain solution (2 mg/mL X-gal, 40 mM citric acid/sodium phosphate, pH 6.0, 5 mM potassium ferrocyanide, 5 mM potassium ferricyanide, 150 mM NaCl, and 2 mM MgCl2) [18], rinsed with PBS, counterstained with eosin, dehydrated, and mounted. SA-β-gal staining was quantified using Image-Pro Plus 5.1 (Media Cybernetics, Bethesda, MD) and the area of dense blue staining in the whole section was calculated.

### SOD Activity and MDA Content

Kidneys were homogenized in ice-cold 20 mM Tris-HCl buffer (pH 7.4). SOD activity was determined using a commercial kit (Jiancheng Bioengineering Institute, Nanjing, China). The mauve product (nitrite) produced by oxidation of hydroxylamine has an absorbance peak at 550 nm. One unit of SOD activity was defined as the amount that reduced the absorbance at 550 nm (A550) by 50%. MDA content was determined with a commercial kit (Jiancheng Bioengineering Institute). According to the manufacturer’s data sheet, the red product has an absorbance peak at 532 nm (A532) [19].

### Microarray Analysis

Total RNA was extracted from kidney samples using Trizol Reagent according to the protocol (Invitrogen, Carlsbad, CA). RNA quality was checked by Northern blotting. Microarray analysis was performed by CapitalBio Corp. (Beijing, China) using rat genome-wide oligonucleotide microarrays according to the methods described previously [20], [21]. Briefly, a Rattus norvegicus genome oligonucleotide set (from Operon oligo database, Rat Genome version 3.0.5, details was opened in http://www.Operon.com), consisting of 26962 5′ amino acid modified 70-mer probes representing 22012 genes and 27044 gene transcripts, was purchased from Operon (Huntsville, AL) and printed on glass slides using a SmartArray™ microarrayer (CapitalBio). Aliquots of 5 µg DNase-treated total RNA were prepared and fluorescent dye (Cy5 and Cy3-dCTP)-labeled cDNA was produced using Eberwine’s linear RNA amplification method [22], and followed by hybridization. Finally, arrays were scanned with a confocal LuxScan™ scanner (CapitalBio), and the data were extracted with SpotData software (CapitalBio). Space- and intensity-dependent normalization based on the LOWESS program was employed [23]. Genes with a Cy3 or Cy5 signal intensity greater than 800 were considered as expressed. For each sample, two hybridizations were performed using a reversal fluorescent strategy. Those genes for which the expression patterns remained consistent in both arrays and the mean expression ratios averaged above twofold were selected as differentially expressed genes.

### Quantitative Real-time PCR

To confirm the microarray results, four representative genes (SIRT-1, connexin43, Irak2 and M6pr) were analyzed by quantitative real-time PCR, according to a modified method [20], cDNA was prepared from 2 µg DNase-treated total RNA from each sample using a First Strand SuperScript II Kit (Invitrogen). Quantitative real-time PCR was performed using a DNA Master SYBR Green I Kit and LightCycler (Roche Diagnostics, Mannheim, Germany), in accordance with the protocols, and the results were analyzed using LightCycler software version 3.5 (Roche Diagnostics). Single PCR products were further verified by melting curve analysis and 1.2% agarose gel electrophoresis. Each gene of interest was normalized relative to β-actin. In addition, each amplification reaction was performed in duplicate, and the mean value was calculated. The mathematical model reported by Pfaffl [24] was employed to analyze the relative expression ratios of these genes. Primers used for the quantitative real-time PCR are listed in Table 1.

**Table 1:** Genes selected and primer choices fo*r qRT–PCR.*

*GB ac*cession no.	Gene symbol		Sequence (5′to 3′)	Amplification size
NM_012567	Cx43	F	TCTGAGTCCTCCACATAGCG	278
		R	GAAGGCACAAAGGTGAGACA	
NM_001107627	SIRT1	F	AATATCCTTTCAGAACCACCAA	173
		R	AGGCGAGCATAAATACCATCT	
NM_001025422	Irak2	F	TACGGGCTCATCTTCTAATACC	80
		R	CCATAACAGAAGAGGGGACAC	
BC079226	M6pr	F	GTTGGGAGAATCAACGAGACTC	104
		R	CGCTGTTCTTTGCCACAGTG	
NM_031144.2	Actin	F	GTACCCAGGCATTGCTGACA	169
		R	CTCCTGCTTGCTGATCCACATC	

### Statistics

All data are presented as mean ± SD. SPSS 11.0 was used to determine statistical significance. Variables were analyzed using a one-way ANOVA, once the difference found, a Tukey-Kramer multiple comparisons test was used to compare among groups. Differences were considered as statistically significant when p was less than 0.05. LuxScan 3.0 image analysis software and Significant Analysis of Microarray (SAM) software were used to analyze the microarray data [25]. CapitalBio® Molecule Annotation System V4.0 (MAS) was used to annotate and analyze the differentially expressed genes.

## Results

Senior rats had slightly higher systolic blood pressure (SBP; mmHg) than young rats, and this was not affected by transplantation (Table 2). The S-Y group had a slightly higher GFR than the S-S group, and the Y-S group had a slightly lower GFR than the Y-Y group, but the differences were not significant (Table 2).

**Table 2:** Body weight, kidney weight, systolic blood pressure (SBP), glomerular filtration rate (GFR), glomerulosclerosis index, tubulo-interstitum scores, SA-β-gal positive rate, superoxide dismutase (SOD) activity and malondialdehyde (MDA) content in the different groups.

	Y	Y-Y	Y-S	S	S-S	S-Y
Body weight (g)	341.9±21.4	329.6±13.3	320.0±20.7	523.9±26.2*	511.5±22.7*	527.4±24.8*
Kidney weight (g)	0.93±0.02	0.92±0.02	0.92±0.02	1.20±0.06*	1.23±0.06 *	1.21±0.07*
SBP (mmHg)	104.6±1.7	105.3±2.4	104.2±2.1	109.2±1.9	109.1±2.2	107.8±1.5
GFR (mL/min)	1.39±0.21	1.35±0.19	1.29±0.25	1.30±0.31*	1.27±0.23*	1.32±0.24*
Glomerulosclerosis index	0.05±0.00	0.05±0.01	0.06±0.00	0.29±0.03*	0.33±0.02*	0.31±0.02*
Tubulo-interstitum scores	0.08±0.07	0.23±0.08&	0.30±0.05&	0.65±0.10 *	0.97±0.17&	0.87±0.11&
SA-β-gal positive rate (%)	0.03±0.01	0.30±0.09*	0.42±0.07* §	4.48±1.39*	6.13±1.34* &	4.63±0.80* $
SOD (U/mg protein)	48.6±7.5	43.5±6.2	36.8±3.6* §	35.4±3.5*	29.4±4.1* &	43.1±5.7* $
MDA (nmol/mg protein)	1.82±0.41	2.03±0.52	2.65±0.46* §	2.73±0.48*	3.64±0.51* &	2.21±0.47* $

* ,P<0.01, vs Y;

§ ,P<0.05, vs YY;

& ,P<0.05, vs S;

$ ,P<0.05, vs SS.

**Figure 1:**
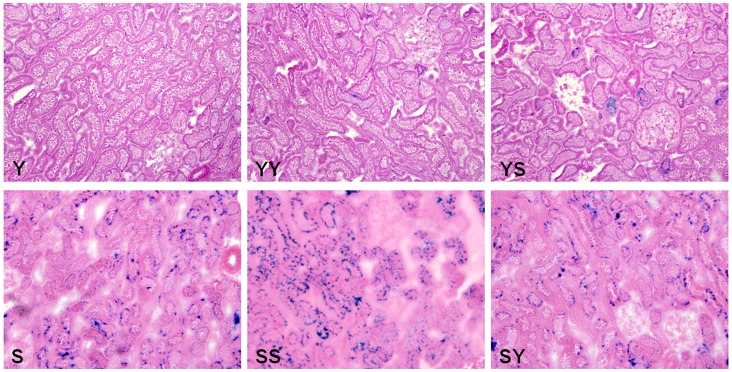
SA-β-gal staining of kidney tissues (×100).

SA-β-gal is a simple biomarker of replicative senescence. The positive rate of staining for SA-β-gal was significantly higher in senior compared to young kidneys, and levels were significantly higher in the Y-S group than in the Y-Y group; in addition, levels were significantly lower in the S-Y group compared to those in the S-S group (Fig. 1, Table 2).

Senior rats had lower SOD activity and higher MDA content than young rats (Table 2). SOD activity was significantly lower and MDA content significantly higher in the Y-S group than in the Y-Y group (Table 2). However, SOD activity was significantly higher and MDA content significantly lower in the S-Y group than in the S-S group (Table 2).

### Histopathology

**Figure 2:**
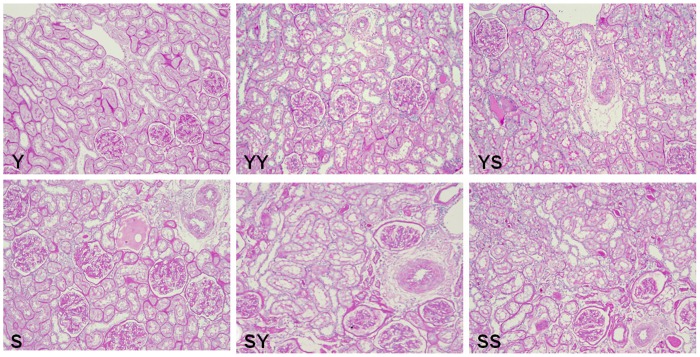
Periodic acid Schiff (PAS) staining of kidney tissues (×100).

Young sham-operated rats had normal renal histology, but the kidneys of senior sham-operated rats had age-related changes (Fig. 2) with glomeruli showing various degrees of glomerulosclerosis, mesangial matrix expansion, and thickening of the glomerular capillary basement membrane. There was also pronounced tubulointerstitial injury, with some areas showing tubular dilation, cast formation, tubular atrophy, thickening, splitting of tubular basement membranes, widening of the interstitium with fibrosis, and loss of focal peritubular capillaries. Transplanted kidneys showed no significant differences in glomerulosclerosis index compared to age-matched controls but had significantly greater tubule-interstitium scores (P<0.05) (Table 2).

### Renal Transcriptional Changes with Age

There were 548 differentially expressed transcripts in young and senior rat kidneys, including 36 that were upregulated and 512 downregulated (Table 3). Using the Kyoto Encyclopedia of Genes and Genomes (KEGG), pathway analysis showed that the genes were clustered in the following pathways: Mitogen-activated protein kinase (MAPK) signaling pathway (8/218, the first number indicates the number of genes changed in this pathway, and the second number how many genes are involved in this pathway), tight junction (7/110), and insulin signaling pathway (6/113) (Tables 3&4&5).

**Table 3:** Gene expression profile in the different pathways among groups.

		S vs Y	Y-S	S-Y
Total gene expression change		548	492	1244
Up-regulation/down-regulation		36/512	127/365	680/574
Up-regulated pathways	MAPK		cPDA2, ATF-2	TGFB, MEKK1, GADD153, MKP,cPDA2, HSP72, c-Myc, ARRB
	insulin signaling			AMPK, FOXO1, GSK-3β,4EBP1, HSL, PHK
Down-regulated pathways	MAPK	Rap1, ERK, MKP, OLK, ATF-2,CASP, MST1/2, TGFB	ERK, TGFB, c-JUN,Cdc42/Rac	
	tightjunction	Claudins, PALS1, Gα, VAP33,JAM, PP2A, MAGI2/3		
	insulin signaling	AMPK, ACC, G6PC, PP1,ERK1/2, eIF4E	G6PC, PHK,ERK1/2	G6PC, eIF4E, PEPCK

**Table 4:** Gene up/down-regulation in the young kidneys transplanted into the senior recipient (YS vs YY) compared with the young recipient.

Up/down	Name	Definition	description	GB accession
Up-regulation	Ehd4	EH-domain containing 4	GTP binding; guanyl nucleotide binding	BC083175
	Atn1	atrophin 1	Neurodegenerative Disorders; system development; nervous system development; Dentatorubropallidoluysian atrophy (DRPLA)	U31777
	Matn1	matrilin 1, cartilage matrix protein	extracellular matrix; extracellular matrix	BC083869
	Cfb	complement component 2	chymotrypsin activity; serine-type peptidase activity; response toexternal biotic stimulus; response to pest, pathogen or parasite;trypsin activity; extracellular region; response to external stimulus;serine-type endopeptidase activity; peptidase activity	BC070923
	Itgb7	integrin, beta 7	Cell adhesion molecules (CAMs); Regulation of actin cytoskeleton; cell adhesion;Focal adhesion; integral to plasma membrane; intrinsic to plasma membrane;Integrin-mediated cell adhesion KEGG; ECM-recipient interaction	AF003598
Down-regulation	Ptk9	protein tyrosine kinase 9	actin binding; cytoskeletal protein binding; kinase activity	BC086536
	Vapa	vesicle-associated membrane protein, associated protein a	Rn-Circadian-Exercise; structural molecule activity; Tight junction	BC061875
	Eif1a	eukaryotic translationinitiation factor 1A	be required for maximal rate of protein biosynthesis	NM_001008773
	Ddx19	DEAD (Asp-Glu-Ala-Asp) box polypeptide 19a	plays a role in zinc induced apoptosis; ATPase activity, coupled; ATPase activity	BC079094
	Nox4	NADPH oxidase 4	oxidoreductase activity; electron transport	AY027527
	Dab2	disabled homolog 2	cellular morphogenesis; morphogenesis; negative regulation of cellular physiological process; negative regulation of cellular process	U95177
	F13b	coagulation factor XIII,beta subunit	Steroid Biosynthesis; Complement and Coagulation Cascades	CO563315
	Sgk	serum/glucocorticoidregulated kinase	protein serine threonine kinase activity;apoptosis; Insulin Signaling	L01624
	Gatm	glycineamidinotransferase	Urea cycle and metabolism of amino groups; mitochondrion;Urea cycle and metabolism of amino groups; Arginine and prolinemetabolism; Glycine, serine and threonine metabolism	BC081785
	Kif21a	kinesin family member 21A	microtubule cytoskeleton; cytoskeleton organization and biogenesis; motor activity	XM_217022
	G6pc	glucose-6-phosphatase	phosphoric monoester hydrolase activity; cytosol; energy derivation by oxidation of organic compounds; endoplasmic reticulum; peptidase activity; Proteasome Degradation; phosphoric ester hydrolase activity; Adipocytokine signaling pathway; Starch and sucrose metabolism; Glycolysis and Gluconeogenesis; Insulin signaling pathway; Galactose metabolism; Glycolysis/Gluconeogenesis	BC090067
	Lgals8	lectin, galactose binding, soluble 8	sugar binding; carbohydrate binding	BC072488
	Nqo2	NAD(P)H dehydrogenase, quinone 2	oxidoreductase activity; electron transport	BC079157
	Dr1	down-regulator oftranscription 1	Transcription	BC083822
	Tmem86a	transmembraneprotein 86A	Ribosome; Celera Assembly; genome assembly; Cytogenetic	XM_218592
	Atf2	activating transcriptionfactor 2	MAPK signaling pathway; system development;nervous system development; MAPK signaling pathway; p38 MAPK signalingpathway; Smooth muscle contraction; transcription	U38938
	Hsd17b1	Hsd17b1	lipid biosynthesis; metabolism; oxidoreductase activity; Steroid Biosynthesis;Androgen and estrogen metabolism; cytoplasm	BC086365
	Frg1	similar to FSHDregion gene 1	transporter activity; binding	CF977214
	Gtf2f2	general transcriptionfactor IIF,polypeptide 2	RNA transcription Reactome; positive regulation of cellular process; Basal transcription factors; transcription; positive regulation of cellular physiological process	D10665
	Agtr1a	angiotensin II recipient,type 1 (AT1A)	Calcium signaling pathway; Neuroactive ligand-recipient interaction; rhodopsin-like recipient activity; GPCRDB Class A Rhodopsin-like; Peptide GPCRs	BC078810
	M6pr	cation-dependentmannose-6-phosphaterecipient	sugar binding; carbohydrate binding	BC079226
	Irak2	interleukin-1 recipient -associated kinase 2	Apoptosis; protein serine threonine kinase activity; protein-tyrosine kinase activity	NM_001025422
	Car12	carbonic anyhydrase 12	lyase activity	XM_343416
	Snapc2	small nuclear RNAactivating complex,polypeptide 2	Transcription	BC079077

**Table 5:** Gene up/down-regulation in the senior kidneys while transplanted into the senior recipient compared with the young recipient.

Up/down	Name	Definition	description	GB accession
Up-regulation	Matn1	matrilin 1, cartilage matrix protein	extracellular matrix; extracellular matrix	BC083869
	Cfb	complement component 2	chymotrypsin activity; serine-type peptidase activity; response to external biotic stimulus; response to pest, pathogen or parasite; trypsin activity; extracellular region; response to external stimulus; serine-type endopeptidase activity; peptidase activity	BC070923
	Mcm6	mini chromosome maintenance deficient 6 (S. cerevisiae)	ATPase activity; cell cycle; transcription; ATPase activity, coupled	U17565
	V1ra13	vomeronasal 1 recipient, a13	rhodopsin-like recipient activity	AY510286
Down-regulation	Sdfr1	stromal cell derived factor recipient 1	sugar binding; carbohydrate binding	X99338
	Dek	DEK oncogene	RNA binding; secretory pathway; GTP binding; guanyl nucleotide binding; intracellular protein transport; secretion	BC079344
	Serinc1	serine incorporator 1	RNA binding	AY539942
	Mat2a	Methionine adenosyltransferase II	magnesium ion binding; selenoamino acid metabolism; methionine metabolism	BC062394
	Golph3	golgi phosphoprotein 3	Golgi stack; Golgi apparatus	AF311954
	Adss	adenylosuccinate synthetase 2	ligase activity, forming carbon-nitrogen bonds; GTP binding; guanyl nucleotide binding	XM_222946
	Cyp2c	Cytochrome P450, subfamily IIC (mephenytoin 4 -hydroxylase)	iron ion binding; electron transport; tetrapyrrole binding; membrane fraction; heme binding; cell fraction; endoplasmic reticulum	BC088146
	Ssr3	signal sequence recipient, gamma	secretory pathway; intracellular protein transport; endoplasmic reticulum; secretion; organelle membrane	BC062015
	TM9SF3	transmembrane 9 superfamily member 3	transporter activity	XM_220013
	Cept1	choline/ethanolamine phosphotransferase 1	lipid biosynthesis	BC079471
	Arl8b	ADP-ribosylation factor-like 8B	GTP binding; guanyl nucleotide binding; intracellular protein transport; small GTPase mediated signal transduction	BC081947
	Pcaf	p300/CBP-associated factor	Notch signaling pathway	NM_001024252
	Dnajc10	DnaJ (Hsp40) homolog, subfamily C, member 10	electron transport; protein folding; electron transporter activity	XM_215751
	Pls3	plastin 3	actin binding; cytoskeleton organization and biogenesis; cytoskeletal protein binding	X70706
	Arl6ip2	ADP-ribosylation factor-like 6 interacting protein 2	GTP binding; guanyl nucleotide binding; response to biotic stimulus	XM_216629
	Eif4e	eukaryotic translation initiation factor 4E	RNA binding; translation Factors; mTOR signaling pathway; insulin signaling pathway; translation; Hypertrophy model; cytoplasm; Insulin Signaling	BC087001
	Kif21a	kinesin family member 21A	microtubule cytoskeleton; cytoskeleton organization and biogenesis; motor activity	XM_217022
	Lgals8	lectin, galactose binding, soluble 8	sugar binding; carbohydrate binding	NM_053862
	Gdi2	GDP dissociation inhibitor 2	protein transport; GTPase regulator activity; vesicle-mediated transport	BC061767
	Igfbp1	insulin-like growth factor binding protein 1	extracellular region; cellular morphogenesis; morphogenesis; Smooth muscle contraction	BC078889
	Zmpste24	zinc metalloproteinase, STE24 homolog	metallopeptidase activity; adipogenesis	XM_233483
	Prkaa1	protein kinase, AMP-activated, alpha 1 catalytic subunit	fatty acid Synthesis; regulation of apoptosis; lipid biosynthesis; mTOR signaling pathway; energy derivation by oxidation of organic compounds; Insulin signaling pathway; protein serine threonine kinase activity; positive regulation of cellular process; negative regulation of cellular physiological process; negative regulation of cellular process; regulation of autophagy; adipocytokine signaling pathway; apoptosis; insulin signaling; regulation of programmed cell death; positive regulation of cellular physiological process	U40819
	Hnrpk	heterogeneous nuclear ribonucleoprotein K	mRNA processing reactome	D17711
	Gphn	Gephyrin	cytoskeleton; coenzyme metabolism; intracellular protein transport; cytoskeletal protein binding	X66366
	Mar-05	membrane-associated ring finger	ligase activity, forming carbon-nitrogen bonds; acid-amino acid ligase activity; ubiquitin cycle; ubiquitin-protein ligase activity; ubiquitin ligase complex; protein ubiquitination	XM_215286
	Ptprc	protein tyrosine phosphatase, recipient type, C	phosphoric ester hydrolase activity; phosphoric monoester hydrolase activity	NM_001109890
	Rgs1	regulator of G-protein signaling 1	signal transducer activity; negative regulation of cellular process; calcium regulation in cardiac cells; smooth muscle contraction	NM_019336
	Cd86	CD86	cell adhesion molecules (CAMs);Toll-like recipient signaling pathway; inflammatory response pathway; type I diabetes mellitus	NM_020081
	M6pr	cation-dependent mannose-6-phosphate recipient	sugar binding; carbohydrate binding	BC079226
	Irak2	interleukin-1 recipient -associated kinase 2	apoptosis; protein serine threonine kinase activity; protein-tyrosine kinase activity	NM_001025422
	Dnaja4	DnaJ (Hsp40) homolog, subfamily A, member 4 (predicted)	protein folding	BC082010
	Id2	Inhibitor of DNA binding 2, dominant negative helix-loop-helix protein	positive regulation of cellular process; development; regulation of progression through cell cycle; TGF-beta signaling pathway; positive regulation of cellular physiological processes	BC086391

In the kidney transplantation groups, the internal milieu of the senior recipients caused upregulation of 127 and downregulation of 365 transcripts in young grafted kidneys (table 4). The KEGG showed that the genes were clustered in the following pathways: MAPK signaling (6/218) and insulin signaling (3/113) (Table 3).

The internal milieu of the young recipient rats upregulated 680 transcripts and downregulated 574 transcripts in senior grafted kidneys (table 5). Using the KEGG, pathway analysis showed that the genes were again clustered in the MAPK signaling (8/218) and insulin signaling (9/113) pathways (Table 3).

### Real-time PCR Analysis of Selected Genes

The PCR results confirmed the expression of the four genes tested in the gene array analysis (Table 6), SIRT-1, Connexin43, Irak2 and M6pr. SIRT-1 expression decreased significantly in young kidneys transplanted into senior recipients, and increased significantly in senior kidneys transplanted into young recipients. Connexin43 gene expression and gap junctions were significantly downregulated, and gap junctional intercellular communication activity was reduced, in old compared to young glomerular mesangial cells (GMCs). Irak2 and M6pr expression were not only consistent with changes in the natural aging process in young kidneys transplanted into senior recipients, but also showed reverse trends in senior kidneys transplanted into younger rats.

**Table 6:** Gene expression in each group vs sham young/senior rats.

		Y-S/Y	S/Y	S-Y/S
SIRT-1	Microarray	−1.1	−1.6	1.1
	RealTime PCR	−1.8	−1.3	8.3
Connexin43	Microarray	−1.1	−1.1	−1.7
	RealTime PCR	−1.6	−1.3	−2.9
Irak2	Microarray	−1.6	−2.1	2.3
	RealTime PCR	1.2	−1.3	4.58
M6pr	Microarray	−1.1	−2.1	1.6
	RealTime PCR	−1.1	−1.3	2.6

## Discussion

The main findings of our study were that: 1. kidney aging occurred in this KT model; 2. aging itself did not influence renal function after KT; 3. accumulation of oxidative stress may be related to the aging effects observed; 4. the change in gene expression profile was different in young and old rat kidneys; these changes were apparent in different pathways: energy metabolism, extracellular matrix (ECM), immunological inflammation, proliferation, differentiation, and apoptosis; 5. the p53-p21, MAPK and insulin signaling pathways may be involved in regulation of these responses. Moreover, Irak2 and M6pr were sensitive renal aging-associated genes.

Kidney aging was present in this KT model because the positive rate of staining for SA-β-gal was significantly higher in senior compared to young kidneys. SA-β-gal is a simple biomarker of replicative senescence [26], [27], and levels were significantly higher in the Y-S group than in the Y-Y group; in addition, levels were significantly lower in the S-Y group compared to those in the S-S group.

Kidney aging was not associated with significant changes in the GFR in our model, but there was a small change in the tubular mesenchymal score, suggesting a potential role of tubular pathogenesis in long-term ischemia/reperfusion injury. Our data are similar to some clinical observations, in which donor-recipient age difference was not associated with increased patient death, death-censored graft failure or serum creatinine at 5 or 10 years, nor was it associated with an increased risk of acute rejection within the first 6 months after KT [28]. However, other clinical studies have shown that donor age, recipient age or donor-recipient age difference was associated with worse short-term or long-term outcomes after KT [3]–[10]. According a recent observational study including 6,317 KT patients, graft outcomes from recipients of older living donor kidneys were inferior to those obtained with younger living donors [29]. These differences may be explained by the different patient populations included and the different criteria used to evaluate outcomes.

In cross-transplantation, the internal milieu in the young rats was associated with increased SOD activity and decreased MDA content in the grafted senior kidneys, whereas the milieu in the older rats had the reverse effect on the grafted young kidneys. SODs catalyze the conversion of (O_2_•−) to H_2_O_2_, which may participate in cell signaling. In addition, SODs play a critical role in inhibiting oxidative inactivation of nitric oxide, thereby preventing peroxynitrite formation and endothelial and mitochondrial dysfunction [30]. Malondialdehyde (MDA) is an end-product of lipid peroxidation and a side product of thromboxane A(2) synthesis. Moreover, it is a frequently measured biomarker of oxidative stress, but its high reactivity and toxicity underline the fact that this molecule is more than “just” a biomarker [31]. Our observations indicate that the oxidative stress associated with aging may be ameliorated by the younger milieu. Free radicals injure proteins, DNA, biological membranes, mitochondria, and accelerate aging in vivo and in vitro [32]. Mitochondria are not only the main site of energy metabolism but also a major source of reactive oxygen species (ROS) [33]. Mitochondrial function and morphology are impaired during aging, with a reduction in SOD and an increase in MDA [34], [35]. These changes result in the accumulation of oxidative stress with aging, rendering the mitochondria of older animals more susceptible to oxidative injury [36].

Compared with young rats, genes in the older rats were largely downregulated, in a manner similar to that seen in humans [37]. Downregulation of the majority of genes indicates that various physiological functions decline in the rat kidney during the aging process. There were significant differences in gene expression between Y-S and S-Y groups, mostly involving the following pathways: Energy metabolism, extracellular matrix (ECM), immunological inflammation, proliferation, differentiation, and apoptosis. These pathways are very important during aging, and are functionally decreased during the aging process [38]–[40]. MAPKs are highly conserved serine/threonine kinases that are activated in response to a wide variety of stimuli and play a role in numerous cell functions including survival, growth, and proliferation [41]. Ito et al. demonstrated that p38 regulates senescence and p16INK4A in hematopoietic stem cells [42]. Zhang et al. showed that inhibiting the p38 pathway mitigates senescence in human umbilical cord blood Endothelial Progenitor Cells (EPCs) [43]. The insulin pathway mediates glucose intolerance, insulin resistance, and type 2 diabetes associated with aging, leading causes of morbidity and mortality, as well as increasing the risk of multiple other age-related diseases, such as cancer, stroke, cardiovascular diseases, Parkinson’s disease, and Alzheimer’s disease [44].

Genes known to be associated with aging, such as SIRT-1, connexin 43, and genes expressed consistently with the age of recipients, such as Irak2 and M6pr, were selected for real-time PCR verification. SIRT-1 expression was significantly lower in the Y-S group, and significantly higher in the S-Y group. SIRT1 is critical to energy metabolism and senescence; it may induce the relative silence of chromatin by sensing cell NAD+ level, and is downregulated during aging [45]. Activation of SIRT1 is associated with longevity and the attenuation of metabolic disorders. SIRT1 in the kidney is cytoprotective and participates in the regulation of blood pressure and sodium balance [46]. The different expression of SIRT-1 protein observed in our study indicated that changes in the internal milieu were associated with activation or inactivation of epigenetic factors involved in the ‘accelerated aging’ or ‘reverse aging’ process. From the microarray data, we observed that the acetylation associated genes, such as PCAF, were involved in the effects of the internal environment on the grafted kidney. Hence, SIRT-1 was not acting alone. In addition, Irak2 and M6pr expression were not only consistent with changes in the natural aging process in young kidneys transplanted into senior recipients, but also showed the reverse trends in senior kidneys transplanted into younger rats. Irak2 plays a key role in tumor necrosis factor (TNF) recipient-associated factor-6 (TRAF-6)-mediated nuclear factor-κB (NF-κB) activation [47]. Aging results in dysregulation of both the innate and adaptive arms of the immune response [48], and the NF-κB/REL family of transcription factors plays a central role in coordinating the expression of a wide variety of genes that control immune responses [49]. Therefore, Irak2 may be an important factor in immune senescence. M6pr is a receptor of mannose-6-phosphate and insulin-like growth factor II (IGF-II), and is involved in the sorting of lysosomal enzymes and the clearance of IGF-II, which directly suppresses malignant cell proliferation [50], [51]. As production of lysosomal enzymes declines with age, and as lysosomes engulf increasingly large aggregates of oxidized, glycated, cross-linked proteins, and lipids that are resistant to enzymatic degradation, dysfunctional lysosomes (bloated with indigestible contents) accumulate in cells as lipofuscin granules. In addition, the integrity of the autophagosomal–lysosomal network appears to be critical in the progression of aging, and a progressive increase in intralysosomal concentrations of free radicals and the age pigment, lipofuscin, further diminish the efficiency of lysosomal protein degradation [52]. The decline in M6pr may be associated with aging and may activate the malignant proliferation of cells. Connexin43 is a major connexin in GMCs, and our previous research indicated that connexin43 gene and protein expression, as well as gap junctions, are significantly downregulated, and that gap junctional intercellular communication activity is reduced, in old vs. young GMCs [53]. Both gene chip and real-time PCR analyses verified these genes expression trends.

There are several limitations of our study. First, our animals were healthy animals with their kidneys lack of kidney dysfunction, which is different from the clinical situation of patients with renal failure and other comorbidities. Second, the right native kidneys of the recipients were not removed, which would worsen the I/R injury and acute renal failure of the grafted kidney, and then influence the gene expression profile. Nevertheless, despite these limitations, we believe our results provide insight into possible genetic manipulation in KT patients.

### Conclusions

In summary, kidney graft aging was present in the current model. There was no difference in KT graft function between young and senior kidney cross-transplantation. Oxidative stress played an important role in kidney aging; the gene expression profile was significantly different in the Y-S compared to the S-Y groups, with differences found largely in the MAPK and insulin signaling pathways. Further studies are warranted to investigate the aging difference in the kidney dysfunction model after KT and Genetic manipulation may be a potential option of ameliorating kidney aging on KT outcomes.
